# Weight gain and parental self-efficacy in a family-based partial hospitalization program

**DOI:** 10.1186/s40337-022-00634-6

**Published:** 2022-08-08

**Authors:** Jessica L. Van Huysse, James Lock, Daniel Le Grange, Renee D. Rienecke

**Affiliations:** 1 Department of Psychiatry, University of Michigan, Ann Arbor, MI USA; 2 Department of Psychiatry and Behavioral Sciences, Stanford University, Palo Alto, CA USA; 3 Department of Psychiatry and Behavioral Sciences, University of California, San Francisco, CA USA; 4 Department of Psychiatry and Behavioral Neuroscience (Emeritus), The University of Chicago, Chicago, IL USA; 5 Eating Recovery Center and Pathlight Mood and Anxiety Centers, Chicago, IL USA; 6 Department of Psychiatry and Behavioral Sciences, Northwestern University, Chicago, IL USA

**Keywords:** Anorexia nervosa, Weight gain, Self-efficacy, Adolescent, Psychotherapy, Family therapy

## Abstract

**Background:**

Family-based treatment (FBT) is an outpatient therapy, though FBT principles have been incorporated in higher levels of care (e.g., partial hospitalization programs, PHPs). It is unknown how participation in a family-based PHP impacts weight restoration and parental self-efficacy.

**Methods:**

Weight gain and parental self-efficacy were examined in 98 participants with anorexia nervosa or atypical anorexia nervosa during the first five weeks of participation in a family-based PHP. Maternal self-efficacy was assessed using the Parent versus Anorexia Scale.

**Results:**

Significant increases in weight, percent expected body weight (EBW), and maternal self-efficacy were observed, with large effect sizes. During the first five weeks of treatment, patients in the PHP gained an average of 4.5 kg, or 8.3% EBW. Maternal self-efficacy improved within two weeks of treatment.

**Conclusions:**

Findings suggest that family-based PHPs may facilitate rapid weight restoration without decreasing parental self-efficacy. Randomized trials are needed to directly compare family-based PHPs to outpatient FBT and PHPs with alternate treatment approaches, including longer-term follow-up and cost-effectiveness modeling.

## Plain English Summary

Family-based treatment (FBT) was designed as an outpatient treatment. Many treatment programs that offer higher levels of care, such as partial hospitalization programs (PHPs), integrate FBT strategies. It remains unclear how well these PHPs promote weight restoration. It is also possible that PHPs may interfere with parental self-efficacy, or parental confidence to help their child, since the treatment team is much more involved in daily care of the patient. This study examined weight gain and maternal self-efficacy in 98 participants with anorexia nervosa or atypical anorexia nervosa enrolled in a family-based PHP. Findings suggested increases in weight and percent expected body weight in the family-based PHP during the first five weeks of treatment. Maternal self-efficacy improved within two weeks of treatment. This study suggests that family-based PHPs may result in rapid weight increases without decreasing parent self-efficacy.

## Background

Family-based treatment (FBT [1]) is an evidence-based outpatient treatment for children and adolescents with anorexia nervosa (AN), often suggested as a first-line treatment for youth [2]. Efforts have been made to incorporate FBT principles to varying degrees into programs offering higher levels of care, including intensive outpatient settings [3], partial hospitalization/day hospital programs [4–9], and inpatient units [10]. The degree of family involvement varies across programs, but may include daily parental involvement at meals [7, 10], parents choosing meals for their children [7, 8], or receiving FBT sessions in addition to day hospital programming [6, 8]. Outcome data are promising, with family-based partial hospitalization programs (PHPs) showing increases in weight along with improvements in eating disorder symptoms, anxiety, depression [5–9], caregiver burden and parental self-efficacy [5, 7].

There is no evidence to support the use of PHPs over outpatient therapy for adolescent AN. A recent systematic review of levels of care in eating disorder treatment did not identify any clear differences in weight gain for patients with AN who were treated at outpatient versus inpatient levels of care [11]. In a randomized study comparing outpatient treatment to inpatient treatment for adolescent AN, there were no additional benefits found for the use of intensive treatment provided in hospital, in fact, outpatient treatment was more cost effective [12]. However, this study did not involve FBT, and PHPs are less intensive than hospital programs. One study randomly assigned patients with eating disorders to either a day treatment program or to traditional outpatient care and found significantly greater improvements along clinical parameters for the day treatment program participants in the short term [13]. To date, no studies have compared outcomes from manualized FBT to outcomes in a family-based higher level of care program.

Family-based PHPs require a significant time commitment from parents and patients. In the studies described above, average length of stay ranged from 31.7 [7] to 149 days [5]—with some of this variability due to increased lengths of stay in non-US-based programs—and program days were six to ten hours long. Alternatively, manualized FBT generally consists of 20 h-long sessions over the course of 12 months [1], requiring much less of a time commitment from families in terms of attending treatment sessions. Understanding whether the additional investment of resources in treatment required by PHPs results in improved clinical outcome is an important area for study.

In manualized outpatient FBT, participants who respond early to treatment (defined as gaining approximately 2.3 kg in the first four weeks of treatment) are more likely to remit at end-of-treatment than those who do not [14–18]. In family-based PHPs, similar patterns have been observed, though the rate of weight gain needed to predict positive treatment response was more rapid (i.e., approximately 4 kg within the first 4 weeks of treatment) [19, 20]. Thus, one might expect that more intensive forms of treatment, such as PHPs, would lead to weight gain more quickly than outpatient treatment.

In addition to weight gain, parental self-efficacy, conceptualized as parental perception of their ability to effectively manage their child’s eating disorder behaviors, has been investigated as a potential predictor of treatment outcomes in FBT. Indeed, greater increases in parental self-efficacy predicts greater weight gain for adolescents in FBT [21]. Further, in a comparison of parental-self efficacy in FBT versus systemic family therapy, changes in maternal self-efficacy were found to mediate the association between treatment type and weight gain, suggesting that interventions specific to FBT may facilitate weight gain via parental self-efficacy [22]. Thus, enhancement of parental self-efficacy related to feeding their child is considered to be a critical component of FBT.

In addition to manualized FBT, there are other interventions that may promote increases in parental self-efficacy. For example, an FBT-informed brief telephone psychoeducational and supportive intervention for parents who were on a wait list awaiting eating disorder evaluation for their child resulted in increases in parental self-efficacy [23]. Conversely, a parent education and skills workshop offered during the first four weeks of FBT did not predict increases in parental-self efficacy compared to a group receiving only FBT. Families enrolled in the workshop showed greater increases in weight at 4 weeks, but differences in weight restoration did not emerge at week 12 or end of treatment [24].

Most studies of parental self-efficacy discussed thus far have occurred in the context of outpatient interventions. One potential limitation of higher levels of care, despite family involvement, is the possibility that parents will not develop the same degree of self-efficacy as parents receiving outpatient treatment, because parents are not managing their child’s meals and eating disorder behaviors in these programs to the extent that they are in manualized FBT. It is difficult to give parents the message that the treatment team has confidence in the parents’ ability to refeed their child, while simultaneously taking over the treatment process for many hours a week. While it is reassuring that parental self-efficacy within family-based PHPs increases by 3- or 6-month follow-up [5, 25], it is also important to examine parental self-efficacy while enrolled in the treatment program, given the concern that the intensive involvement of staff may undermine parent confidence in the acute phase of treatment. One study examined parental self-efficacy at baseline and two-weeks of family-based PHP, and findings suggested significant increases in parental self-efficacy, but the sample size was small (n = 21) and requires replication [7]. Another study of a four-session FBT-informed intervention for families of youth with AN while medically hospitalized (n = 44) demonstrated significant increases in parental self-efficacy from pre- to post-intervention, again suggesting that FBT-based psychoeducation and collaborative problem-solving with families may promote increases in self-efficacy despite the child requiring a high level of care [26]. However, given the theoretical concerns related to implementing FBT within higher levels of care and small sample sizes, additional studies are needed.

The purpose of the current study was to examine weight gain and parental self-efficacy for adolescents participating in a family-based PHP. We focused on the first five weeks of treatment given evidence of the importance of early change in FBT [14, 15], and the average duration of the PHP stay. The core of the interventions during this time frame was based upon the first phase of FBT. That is, treatment goals in the PHP generally focus on weight gain and support for parental self-efficacy to manage eating disorder behaviors. It was hypothesized that patients participating in the PHP would gain weight rapidly, given the nature of the intensive treatment setting, but that parental self-efficacy would not improve in the early weeks of the PHP.

## Methods

### Participants

Participants were a convenience sample of 98 consecutive admissions to the PHP program who consented to research participation. All PHP participants were between the ages of 12 and 18 (*M *= 15.10, SD = 1.86), and met DSM-5 criteria for AN or atypical AN, with average admission weights below 85% median body mass index (BMI) for age and sex. Most participants identified as female (92.9%). The majority of participants were white (90%), with others identifying as Asian (6%), Hispanic (3%), or Black (1%). On average, duration of illness was nearly one year (*M *= 10.88 months, SD = 9.39), with a range of 1.5 months to 4.5 years.

### Treatment

Patients are referred to the family-based PHP from multiple sources, including from outpatient providers (59.2%), following medical admission (22.4%) or psychiatric admission (9.2%), self-referrals (3.1%), or referral from an emergency department (1%) or residential treatment center (1%). Referral source was unknown for 4.1% of patients. Most patients had some prior outpatient intervention before entry to the PHP (77.6%), including outpatient therapy (60.2%) and/or other outpatient interventions, such as dietitian or adolescent medicine visits (54.1%). Patients admitted to the PHP without an outpatient trial are generally admitted to the PHP level of care due to symptom severity (e.g., BMI < 80%) or lack of access to outpatient care. Many patients were medically hospitalized (39.8%) and/or psychiatrically hospitalized (13.3%) prior to PHP admission. Only 5.1% of patients had no prior treatment. Patients must have a caregiver (usually parent(s)) who are able to participate in the PHP. There are no exclusion criteria for participation in the research study. The purpose of the PHP is to provide families with a strong foundation in Phase 1 of FBT, including facilitating a positive weight trajectory and parental management of meals and other eating disorder behaviors. Following completion of the PHP, patients are typically discharged to an intensive outpatient program (IOP) for 2–3 weeks, followed by outpatient care to complete eating disorder treatment.

Patients in the PHP participate in programming Monday through Friday for 6 h a day. Given that this study focused on the initial five weeks of treatment, patients received approximately 150 h of intervention at the end of the study period. The daily schedule involves two meals and a snack and attendance at therapy groups drawn from treatment approaches including dialectical behavior therapy (DBT), cognitive-behavioral therapy (CBT), and cognitive remediation therapy (CRT), as well as groups addressing topics such as body image and self-expression. Patients are also seen by the program’s adolescent medicine physicians (one visit per week) and psychiatrist (two visits per week). The program is family-based, with an emphasis on Phase one of manualized FBT. Prior to joining the PHP, all families participate in two introductory FBT sessions, following sessions one and two as outlined in the FBT manual [1]. Upon joining the PHP, daily parental involvement in at least one meal is required, and parents are tasked with the responsibility of weight restoration as in manualized FBT. For example, parents select meals and snacks served to their child during the PHP treatment day, even for meals that they may not be present for, by ordering from the hospital menu on behalf of their child. Likewise, parents are tasked with managing eating-related decisions at home. To facilitate development of these skills, parents also attend weekly parent debriefing sessions and a weekly parent skills group. After completion of the PHP, most patients step down to the intensive outpatient program (IOP), with programming three days a week for three hours a day. Patients in IOP have one meal and one snack during the treatment day. See Hoste [7] for further program details.

### Procedure

Patients and families completed questionnaires at entry into the program, two weeks into the program, one month into the program, and at end-of-treatment in the PHP. Weights were taken three times per week. All patients completed questionnaires, and those that were willing to participate in research signed informed consent. Of patients who entered the treatment program, 81.4% consented to participate in the research database, from which participants for the current study were drawn.

### Measures

#### Weight and expected body weight (EBW)

Participants were weighed in light indoor clothing with no shoes. EBW was determined based upon median BMI for age and sex utilizing Centers for Disease Control and Prevention (CDC) growth charts.

#### Parents versus anorexia scale (PVA) [27]

The PVA is a 7-item scale assessing a parent’s sense of self-efficacy in the context of FBT. Specifically, parents are asked to what degree they feel they have the ability to bring about their child’s recovery in the home setting, with items such as, “I feel equipped with specific practical strategies for the task of bringing about the complete recovery of my child in the home setting.” measured on a 5-point Likert scale from “strongly disagree” to “strongly agree”. Higher scores indicate greater self-efficacy. Preliminary studies indicate adequate psychometric properties for the PVA [27], including Cronbach’s alphas above 0.7 and expected correlations with related constructs. The PVA was administered to parents at baseline, two weeks into the program, one month into the program, at end-of-treatment in the PHP (approximately week 6). We examined PVA data at baseline and week two, to assess very early change in PVA scores. We were unable to examine PVA data at additional time points due to high rates of missing follow-up data. Likewise, only data for mothers are presented due to high rates of missing data for fathers.

### Statistical analyses

All analyses were conducted in SPSS version 25 [28]. Baseline weights/EBW data were available for 93.9% of cases, while follow-up (week 5) weight/EBW was available for 80.6% of cases. Missing weights at baseline were due to recording errors. Missing weights at follow-up were due to treatment dropout (n = 11, 58% of missing weights), transitioning to a higher level of care (n = 4, 21%), weight not obtained due to clinic closure or patient absence (n = 2, 10.1%), or discharge due to adequate treatment progress (n = 2, 10.5%). PVA follow-up data were available for 50% of PHP participants, with missing data primarily due to non-returned questionnaires (98%). Multiple imputation with five imputed data sets was used, as this is the recommended approach to managing missing data under most circumstances [29, 30].

Baseline and week five weight and percent EBW are reported, as well as parental self-efficacy at baseline and week two. Paired-sample t-tests and Cohen’s *d* are reported to indicate within-treatment group change for each variable.

## Results

**Fig. 1:**
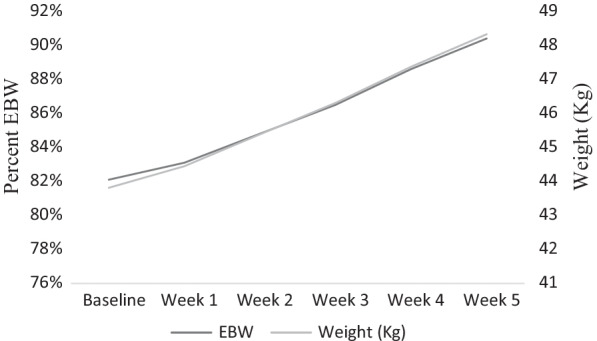
Change in weight (kg) and %EBW during treatment. *Note*: EBW, expected body weight

As shown in Table 1 and Fig. 1, there were significant increases in weight and percent EBW during treatment with large effect sizes. Specifically, patients in the PHP experienced an average increase of 4.5 kg (9.2 lbs), or 8.3% improvement in percent EBW during the first 5 weeks of treatment. Contrary to hypotheses, parental self-efficacy scores improved rapidly by week two in the PHP (Table 1).

**Table 1:** Percent expected body weight and parental self-efficacy at baseline and follow-up

Variable	Baseline M (SD)	Follow-up M (SD)	n	t (*df*)	Cohen’s d
Weight (kg)	43.81 (6.49)	48.33 (6.75)	98	15.40 (155)*	1.70
%EBW	82.1% (7.0%)	90.4% (7.4%)	98	16.31 (2790)*	1.68
PVA	18.51 (3.80)	22.34 (3.70)	84	7.13 (35)*	0.97

Follow-up data are from week 5 for weight and %EBW; week 2 for PVA. Pooled multiple imputation data are presented. Cohen’s *d* demonstrates effect size of baseline to follow-up

EBW, expected body weight; PVA, parent versus Anorexia Scale

*p < .05

## Discussion

This study aimed to examine early changes in weight gain and parental self-efficacy for adolescents and families participating in a family-based PHP. Given the intensive nature of PHP treatment, it was hypothesized that rapid weight gain would be observed in the PHP group. However, it was also expected that the intensive nature of the PHP could interfere with parental self-efficacy due to a perception that extensive staff support is needed to interrupt the eating disorder, resulting in lower parental self-efficacy scores. Findings were suggestive of rapid increases in weight and %EBW. Contrary to hypotheses, parental self-efficacy improved rapidly in the PHP, suggesting that the PHP level of care may not interfere with parental-self efficacy as has previously been hypothesized [7].

Importantly, the particular PHP investigated herein makes explicit attempts not to undermine parental self-efficacy, using methods such as parents choosing foods that are served during the program day rather than prescribed meal plans, deferring to parental judgement during program meals, requiring parent participation in meals, and taking a collaborative stance with parents [7]. Thus, the PHP examined in the current study is designed not to “take over” the treatment process for parents. In the past, clinical observation has led to concerns that self-efficacy may be undermined by common parental behaviors during program enrollment, such as parents stating they are more likely to initially present challenge foods in the program rather than at home, as they feel their child is more likely to eat in the PHP setting. Although these clinical observations have led to the hypothesis that parental self-efficacy may be undermined in the PHP, perhaps instead these opportunities lead to successes in the PHP program that are translated to the home environment and increase parental efficacy. Consistent with this hypothesis, caregiver fear and self-blame have been shown to predict lower parental self-efficacy and more caregiver accommodation/enabling of eating disorder behaviors [31]. Likewise, increases in parental self-efficacy in the context of a two-day group emotion focused family therapy intervention was predicted by decreases in parental self-blame and fear [32]. It is possible that the family-based PHP reduces caregiver fear by providing reassurance that a high level of provider support is available, while also providing skills to translate to the home environment and instill confidence.

The decision to pursue treatment in a family-based PHP must be considered alongside the additional time and financial commitment required by a higher level of care. It is generally considered preferable to treat adolescents initially in the least restrictive level of care possible and to begin with outpatient treatment [33], and it remains unknown whether the extra investment of time and financial resources in family-based PHPs results in more rapid weight gain or other clinical improvements compared to manualized outpatient FBT. Examining prior studies of outpatient FBT, patients have been shown to reach about 91–94% EBW after 6 months of treatment [34]. In another study, patients who demonstrated early weight gain in FBT reached an average of 100% EBW at the end of 12 months of treatment, compared to an average of 93% EBW for those who did not demonstrate this early weight gain [18]. At 12-month follow up, average EBWs of 93–96% have been reported [34]. Together, the average EBW of 90% reached by week 5 in the current study appears encouraging. Similar examination of prior reports of PVA scores suggests a similar pattern of PVA scores in the current study compared to prior research. In one study, the mean PVA score for parents who had positive treatment response (defined as the patient being within 10% of EBW) was 25.0, compared to a mean score of 15.7 for parents on the treatment wait list [27], and another study including FBT participants reported PVA scores of 19.5 at baseline and 23.7 at 4 weeks [24]. Thus, the change from a baseline score of 18.5 to a 2-week PVA score of 22.3 in the current study appears relatively consistent with findings of PVA scores in prior research, though direct comparisons across samples should be made with caution.

Future studies using randomized designs that compare manualized outpatient FBT to a family-based PHP are needed, and should integrate measures of treatment effectiveness, cost-effectiveness, and treatment acceptability. Similarly, adolescent PHP or residential treatment programs that are not explicitly family-based typically have family components (e.g., educational sessions), and it is unknown whether the enhanced level of family involvement in family-based PHPs results in improved outcomes in the short- or long- term. A recent non-randomized study that compared a cohort of medically hospitalized patients who received an FBT-informed intervention while hospitalized, compared to a prior cohort of youth who did not receive the FBT-intervention, suggested that the group receiving the FBT-based intervention were more likely to show positive weight outcomes and had lower rates of re-hospitalization at 3- and 6-months following hospital discharge [26]. This finding is especially interesting because parents were generally very involved in the hospitalization in both groups, meaning that even non-FBT parents were typically staying with the youth for the hospitalization and interacting with providers. Comparisons of programs that offer parental psychoeducation or involvement in therapy sessions (but not FBT) to specific FBT-based interventions are needed, but this study provides some suggestion that FBT-informed interventions facilitate a specific type of parental engagement that predicts positive outcomes upon transition to outpatient care [26].

The study has several limitations. Notably, long-term follow-up data were not available for the PHP patients. Although early weight gain predicts good outcome a year later in manualized outpatient FBT studies, it is not known whether rapid weight gain in the PHP also predicts good outcome a year later, or whether the weight gain trajectory in the PHP predicts better outcomes than FBT long-term. Indeed, given the financial and time commitments associated with a PHP, it could be argued that larger differences than those observed may be expected in order to make the intensity of the intervention worthwhile. For example, PHP participants received 150 h of intervention in 5 weeks, whereas in weekly outpatient FBT, about five hours of intervention would occur in the same time period. Additionally, it is possible that with the step-down to weekly outpatient treatment after leaving the PHP/IOP, patients’ progress may be disrupted, particularly if they transition to a non-FBT provider. However, an examination of the family-based PHP described in this study found that patients’ improvements were maintained at 3-month follow-up [25], suggesting that progress can be sustained after patients step down to less intensive treatment. An additional limitation is that, although family-based programs offering higher levels of care seem to be growing in popularity, the findings of this study refer to a specific and highly family-based PHP, limiting the generalizability of the findings.

Rates of missing data were higher than ideal, particularly for the PVA (~50%), but also for week 5 weight (80.6%), which is a common concern in similar treatment settings [19]. We utilized multiple imputation strategies to manage the missing data, and missing PVA or weight data were not associated with patient age, EDE global scores at baseline, duration of illness, or %EBW at baseline or week 5, or baseline or week 2 PVA. Overall, it appears that data were most likely missing at random (MAR), and multiple imputation models should have reasonably accounted for this [30, 35], though it is possible that results would differ with a more complete data set, particularly if incomplete data were more likely in poor responders to treatment. Overall, improvement in data collection strategies in naturalistic treatment studies are needed. We also focused on examination of PVA scores after two weeks of treatment, when data were most complete. Thus, it is unknown how parental-self efficacy changes with additional time in each treatment, though prior studies have shown that mother PVA scores increase significantly during a PHP, and these gains appear to be maintained at 3-month follow-up (e.g., *M* 3-month follow-up PVA score of 25.1) [25]. Finally, we were unable to examine PVA scores in fathers due to low response rates in the PHP sample. Study procedures for the PHP may have enhanced the rate of missing data in fathers. Specifically, families were given paper follow-up questionnaires to complete during the PHP program day, and mothers are more likely to be the parent in attendance for daily programming, even though fathers may still be highly involved in family sessions and the refeeding process. When a parent was not present on the day the questionnaires were handed out, questionnaires were sent home for the parent who was not present to complete, but they were not always returned. Regardless, the higher rate of missing data for fathers is consistent with research on lower father versus mother involvement in FBT and reinforces the need for continued investigation of the role of fathers in treatment [36]. Additionally, though the PVA has typically been conceptualized as a measure of parental self-efficacy in regard to eating disorder treatment, a recent study demonstrated no association between the PVA and a measure of general self-efficacy [37]. Further research is needed to clarify the construct assessed by the PVA, as when the scale was developed it did demonstrate convergent validity with a measure of external versus internal locus of control [27], suggesting that parents who indicated higher self-efficacy on the PVA also reported more internal locus of control. Nevertheless, this study demonstrated rapid increases in PVA scores among parents, suggesting increases in knowledge or efficacy related to FBT, likely as a result of being immersed in a treatment milieu that supports these concepts.

Lastly, some information was unavailable that would be useful to further characterize the participants or treatment outcomes. For example, though we were able to ascertain the setting(s) in which patients had received prior treatment (e.g., outpatient, medical hospitalization, etc.), specific information about prior treatment modality was not available. Thus, it is unclear what proportion of participants had prior exposure to FBT. Though several characteristics of the sample suggest severe illness in support of the PHP level of care, including low baseline % EBW and high rates of prior medical/psychiatric hospitalization, it is possible that some of the participants would have benefitted from outpatient FBT, and the reason for previous treatment failure was lack of access to FBT, rather than the need for a higher level of care.

## Conclusions

Despite these limitations, this study replicates prior findings supporting rapid weight gain among patients in a family-based PHP, with no decrease in parental self-efficacy observed. Findings indicate that family-based PHPs may be a promising treatment option for those who cannot participate in outpatient FBT. These exploratory findings support future investigations using randomized designs with longer-term follow-up, which should also investigate cost-effectiveness to guide determinations regarding when PHPs may be indicated.

## Data Availability

The dataset used and/or analyzed during the current study are available from the corresponding author on reasonable request.

## Abbreviations

AN: Anorexia nervosa
BMI: Body Mass Index
CBT: Cognitive behavior therapy
CDC: Centers for Disease Control and Prevention
CRT: Cognitive remediation therapy
DBT: Dialectical behavior therapy
EBW: Expected body weight
EDE: Eating disorder examination
IOP: Intensive outpatient program
FBT: Family-based treatment
MAR: Missing at random
PHP: Partial hospital program
PVA: Parent versus Anorexia Scale

## References

[1] Lock J, Le Grange D (2013). Treatment manual for anorexia nervosa: a family-based approach.

[2] Academy for Eating Disorders. A guide to selecting evidence-based psychological therapies for eating disorders. [Internet]. 2020. Available from: https://higherlogicdownload.s3.amazonaws.com/AEDWEB/27a3b69a-8aae-45b2-a04c-2a078d02145d/UploadedImages/Publications_Slider/FINAL_AED_Psycholohgical_book.pdf.

[3] Marzola E, Knatz S, Murray SB, Rockwell R, Boutelle K, Eisler I (2015). Short-term intensive family therapy for adolescent eating disorders: 30-month outcome. Eur Eat Disord Rev..

[4] Easton E, Manwaring J, Salada G, Hartman G, Bermudez O, Johnson C, Murray SB, Anderson LK, Cohn L (2017). Family in residence program: a family empowerment model for higher levels of care. Innovations in family therapy for eating disorders.

[5] Girz L, Robinson AL, Foroughe M, Jasper K, Boachie A (2013). Adapting family-based therapy to a day hospital programme for adolescents with eating disorders: preliminary outcomes and trajectories of change. J Fam Ther..

[6] Henderson K, Buchholz A, Obeid N, Mossiere A, Maras D, Norris M (2014). A family-based eating disorder day treatment program for youth: examining the clinical and statistical significance of short-term treatment outcomes. Eat Disord..

[7] Hoste RR (2015). Incorporating family-based therapy principles into a partial hospitalization programme for adolescents with anorexia nervosa: challenges and considerations. J Fam Ther..

[8] Martin-Wagar CA, Holmes S, Bhatnagar KAC (2019). Predictors of weight restoration in a day-treatment program that supports family-based treatment for adolescents with anorexia nervosa. Eat Disord..

[9] Ornstein R, Lane-Loney S, Hollenbeak C (2012). Clinical outcomes of a novel, family-centered partial hospitalization program for young patients with eating disorders. Eat Weight Disord-St..

[10] Halvorsen I, Reas DL, Nilsen J-V, Rø Ø (2018). Naturalistic outcome of family-based inpatient treatment for adolescents with anorexia nervosa. Eur Eat Disord Rev..

[11] Hay PJ, Touyz S, Claudino AM, Lujic S, Smith CA, Madden S (2019). Inpatient versus outpatient care, partial hospitalisation and waiting list for people with eating disorders. Cochrane Database Syst Rev.

[12] Gowers SG, Clark A, Roberts C, Griffiths A, Edwards V, Bryan C (2007). Clinical effectiveness of treatments for anorexia nervosa in adolescents: randomised controlled trial. Br J Psychiat.

[13] Kong S (2005). Day treatment programme for patients with eating disorders: randomized controlled trial. J Adv Nurs..

[14] Doyle PM, Le Grange D, Loeb K, Doyle AC, Crosby RD (2010). Early response to family-based treatment for adolescent anorexia nervosa. Int J Eat Disorder..

[15] Hughes EK, Sawyer SM, Accurso EC, Singh S, Le Grange D (2019). Predictors of early response in conjoint and separated models of family-based treatment for adolescent anorexia nervosa. Eur Eat Disord Rev..

[16] Le Grange D, Accurso E, Lock J, Agras S, Bryson SW (2014). Early weight gain predicts outcome in two treatments for adolescent anorexia nervosa. Int J Eat Disorder..

[17] Lock J, Couturier J, Bryson S, Agras S (2006). Predictors of dropout and remission in family therapy for adolescent anorexia nervosa in a randomized clinical trial. Int J Eat Disorder..

[18] Madden S, Miskovic-Wheatley J, Wallis A, Kohn M, Hay P, Touyz S (2015). Early weight gain in family-based treatment predicts greater weight gain and remission at the end of treatment and remission at 12-month follow-up in adolescent anorexia nervosa. Int J Eat Disorder..

[19] Brown TA, Murray SD, Anderson LK, Kaye WH (2020). Early predictors of treatment outcome in a partial hospital program for adolescent anorexia nervosa. Int J Eat Disorder..

[20] Van Huysse JL, Smith K, Mammel KA, Prohaska N, Rienecke RD (2020). Early weight gain predicts treatment response in adolescents with anorexia nervosa enrolled in a family-based partial hospitalization program. Int J Eat Disorder..

[21] Byrne CE, Accurso EC, Arnow KD, Lock J, Le Grange D (2015). An exploratory examination of patient and parental self-efficacy as predictors of weight gain in adolescents with anorexia nervosa. Int J Eat Disorder..

[22] Sadeh-Sharvit S, Arnow KD, Osipov L, Lock JD, Jo B, Pajarito S (2018). Are parental self-efficacy and family flexibility mediators of treatment for anorexia nervosa?. Int J Eat Disorder..

[23] Spettigue W, Maras D, Obeid N, Henderson KA, Buchholz A, Gomez R (2015). A psycho-education intervention for parents of adolescents with eating disorders: a randomized controlled trial. Eat Disord..

[24] Ganci M, Pradel M, Hughes EK (2018). Feasibility of a parent education and skills workshop for improving response to family-based treatment of adolescent anorexia nervosa. Int J Eat Disorder..

[25] Rienecke RD, Richmond RL (2018). Three-month follow-up in a family-based partial hospitalization program. Eat Disord..

[26] Matthews A, Peterson CM, Peugh J, Mitan L (2019). An intensive family-based treatment guided intervention for medically hospitalized youth with anorexia nervosa: parental self-efficacy and weight-related outcomes. Eur Eat Disorders Rev.

[27] Rhodes P, Baillie A, Brown J, Madden S (2005). Parental efficacy in the family-based treatment of anorexia: preliminary development of the Parents Versus Anorexia Scale (PVA). Eur Eat Disord Rev..

[28] IBM Corp. Released 2017. IBM SPSS Statistics for Windows, Version 25.0. Armonk, NY: IBM Corp. IBM Corporation, 2015.

[29] Rubin DB (1987). Multiple imputation for nonresponse in surveys.

[30] Van Ginkel JR, Linting M, Rippe RCA, van der Voort A (2020). Rebutting existing misconceptions about multiple imputation as a method for handling missing data. J Pers Assess..

[31] Stillar A, Strahan E, Nash P, Files N, Scarborough J, Mayman S (2016). The influence of carer fear and self-blame when supporting a loved one with an eating disorder. Eat Disord..

[32] Strahan EJ, Stillar A, Files N, Nash P, Scarborough J, Connors L (2017). Increasing parental self-efficacy with emotion-focused family therapy for eating disorders: a process model. Person Centered Exp Psychother.

[33] National Institute for Health and Care Excellence. Eating disorders: Recognition and treatment. NICE guideline (NG69). 2017. https://www.nice.org.uk/guidance/ng69/evidence/full-guideline-pdf-161214767896. 28654225

[34] Le Grange D, Hughes EK, Court A, Yeo M, Crosby RD, Sawyer SM (2016). Randomized clinical trial of parent-focused treatment and family-based treatment for adolescent anorexia nervosa. J Am Acad Child Adolesc Psychiatry.

[35] Asendorpf JB, van de Schoot R, Denissen JJA, Hutteman R (2014). Reducing bias due to systematic attrition in longitudinal studies: the benefits of multiple imputation. Int J Behav Dev..

[36] Hughes EK, Burton C, Le Grange D, Sawyer SM (2018). The participation of mothers, fathers, and siblings in family-based treatment for adolescent anorexia nervosa. J Clin Child Adolesc..

[37] Hamadi L, Hurlock T, Line H, Holliday J (2020). Do parent factors predict early weight gain in family therapy for anorexia nervosa?. Revis Var Commun Treat Sample.

